# Altitude effects on spatial components of vascular plant diversity in a subarctic mountain tundra

**DOI:** 10.1002/ece3.5081

**Published:** 2019-03-22

**Authors:** Lucy Naud, Johannes Måsviken, Susana Freire, Anders Angerbjörn, Love Dalén, Fredrik Dalerum

**Affiliations:** ^1^ Department of Zoology Stockholm University Stockholm Sweden; ^2^ Department of Bioinformatics and Genetics Swedish Museum of Natural History Stockholm Sweden; ^3^ Research Unit of Biodiversity (UMIB, UO‐CSIC‐PA) Oviedo University Mieres Spain; ^4^ Mammal Research Institute, Department of Zoology and Entomology University of Pretoria Hatfield South Africa

**Keywords:** alpha diversity, alpine, altitude gradient, beta diversity, community structure, flora, modularity, nestedness, plants, tundra

## Abstract

Environmental gradients are caused by gradual changes in abiotic factors, which affect species abundances and distributions, and are important for the spatial distribution of biodiversity. One prominent environmental gradient is the altitude gradient. Understanding ecological processes associated with altitude gradients may help us to understand the possible effects climate change could have on species communities. We quantified vegetation cover, species richness, species evenness, beta diversity, and spatial patterns of community structure of vascular plants along altitude gradients in a subarctic mountain tundra in northern Sweden. Vascular plant cover and plant species richness showed unimodal relationships with altitude. However, species evenness did not change with altitude, suggesting that no individual species became dominant when species richness declined. Beta diversity also showed a unimodal relationship with altitude, but only for an intermediate spatial scale of 1 km. A lack of relationships with altitude for either patch or landscape scales suggests that any altitude effects on plant spatial heterogeneity occurred on scales larger than individual patches but were not effective across the whole landscape. We observed both nested and modular patterns of community structures, but only the modular patterns corresponded with altitude. Our observations point to biotic regulations of plant communities at high altitudes, but we found both scale dependencies and inconsistent magnitude of the effects of altitude on different diversity components. We urge for further studies evaluating how different factors influence plant communities in high altitude and high latitude environments, as well as studies identifying scale and context dependencies in any such influences.

## 1 INTRODUCTION

Environmental gradients, gradual changes in abiotic factors which affect species abundances and distributions, can be very important for the spatial distribution of biodiversity (Gaston, 2000). Studies which focus on quantifying biodiversity along environmental gradients are important for improving our understanding of how species communities respond to local environmental conditions (Whittaker, Willis, & Field, 2001), and can also be informative for our understanding of how species communities evolve (Emerson & Gillespie, 2008). Many environmental gradients are linked to primary productivity through access to water and net influx of energy (Hawkins et al., 2003). For instance, the latitude gradient along which species richness decreases from the tropic to polar regions is largely thought to be caused by primary productivity (Currie, 1991), although alternative explanations, such as species–area relationships or geometric constraints, exist (Willig, Kaufman, & Stevens, 2003).

It has long been recognized that species richness shifts in predictable ways with increasing altitude in a wide range of organisms (Lomolino, 2001). As with other environmental gradients, understanding the ecological processes associated with altitude gradients can be fundamental for our ability to develop and test more general theories regarding spatial biodiversity patterns (Brown, 2001). Mountains are also interesting from a fragmentation perspective (Steinbauer et al., 2016), since they are surrounded by areas of lower elevations and hence can be regarded as a special case of ecological islands (MacArthur, 1972). High altitude environments are, like other areas with extreme climates, also expected to experience larger biotic effects of global warming than temperate areas (Beniston, 2003; Grabherr, Gottfried, & Pauli, 1994). However, although abiotic factors such as temperature and precipitation predictably change with altitude (Currie et al., 2004), the corresponding change in biodiversity is not as uniform and we currently have a limited understanding of how altitude influences biodiversity across different spatial and taxonomic scales (Rahbek, 2005).

Arctic plants face several growth constraints caused by environmental conditions, many of which mimic the constraints faced by plants in high‐alpine environments (Billings & Mooney, 1968). Subsequently, in arctic conditions, any altitude‐mediated effects on diversity are expected to be amplified, with a subsequent accentuation of the altitude diversity gradient (Chapin & Körner, 1995). This is particularly relevant in the face of ongoing climate change, which is occurring twice as fast in the Arctic than as in many other environments (Anasimov et al., 2007). Growth, reproduction, seedling development and survival are hampered by persistent snow cover during winter and by low temperatures, reduced period for growth and low availability of soil nutrients during summer (Bliss, 1956, 1971; Sørensen, 1941; Wilson, 1996). However, although many of these constraints may be released by a warmer climate, altitude variation of plant diversity at high latitudes is complex with multiple drivers, many which occur at very local scales (Callaghan et al., 2013).

Whittaker (1960) proposed a partitioning of diversity into alpha, beta, and gamma components, which describes patterns of diversity from very local (alpha) to landscape (gamma) scales. Although these spatial diversity components describe variation within and among biological communities, they do not fully describe patterns of species distributions across time and space (Dalerum, de Vries, Pirk, & Cameron, 2017). For instance, both spatially nested and modular species distributions could theoretically result in similar values of all three diversity components compared to antinested or completely random patterns of variation. Since both nested and modular patterns of the spatial distributions of species may be highly relevant for ecosystem properties and our management of them (Bastolla et al., 2009; Patterson & Atmar, 1986), metacommunity structure has become a central component of modern community ecology (Leibold et al., 2004). In a fully nested pattern of species distributions, all species that are present in species‐poor locations are also present in species‐rich ones (Galeano, Pastor, & Iriondo, 2009). In reality, fully nested patterns are rare, but communities in which species‐rich locations contain more unique species than species‐poor ones are commonly observed (Wright, Patterson, Mikkelson, Cutler, & Atmar, 1998). Modularity describes the extent to which species interactions are clustered so that species are more ecologically associated within than across modules. It is expected to be an important property of ecological communities (Olesen, Bascompte, Dupont, & Jordano, 2007). In a spatial context, modular distribution structures are the consequence of species turnover (Baselga, 2010), which has been suggested as important for biodiversity maintenance (Chesson, 2000). Since, modular structures represent fundamentally different processes behind community structuring compared to nestedness (Williams, 1996), it is important to evaluate the relative prevalence of each structure for a complete understanding of spatial variation in diversity (Gaston & Blackburn, 2000).

In this study, we quantified how vegetation cover, species richness, species evenness, and beta diversity of vascular plants changed along altitude gradients in a subarctic mountain tundra in northern Sweden. We also tested for nested and modular patterns of community composition, and if these corresponded to altitude. Such quantifications of how biodiversity shifts with altitude are paramount for our ability to form testable hypothesis regarding the processes underlying spatial biodiversity variation (Dalerum, Retief, Havemann, Chimimba, & Rensburg, 2019). At northern latitudes, with the amplified effects of global warming (Anasimov et al., 2007), mechanistic understanding is particularly important for our abilities to manage ecosystems under environmental change (Waide et al., 1999). However, we would like to highlight that this study focuses on quantifying patterns of biodiversity change rather than trying to relate any shifts in biodiversity to potential drivers. We have chosen this approach since we believe that a proper identification of ecological patterns is necessary before any mechanistic hypotheses are developed to test the underlying processes behind such patterns (Dalerum, 2012). Although intuitive, a monotonic decline in species richness and diversity with increasing altitudes is not generically supported by empirical observations (Colwell & Lees, 2000). Instead, both a hump‐shaped relationship between altitude and species richness, with a maximum diversity at midaltitudes and a pattern with essentially constant diversity from low to midaltitude followed by a strong decline at the highest elevations have also been observed (Rahbek, 1995). However, patterns of community composition have not yet been quantified. Hence, it is unclear to what extent alterations in species communities with varying altitudes are caused by species turnover, which would generate modular patterns, or a dilution of species that are poorly adapted to specific altitude bands, which may give rise to nested or partially nested patterns of community composition.

## 2 METHODS

### 2.1 Study area

**Figure 1:**
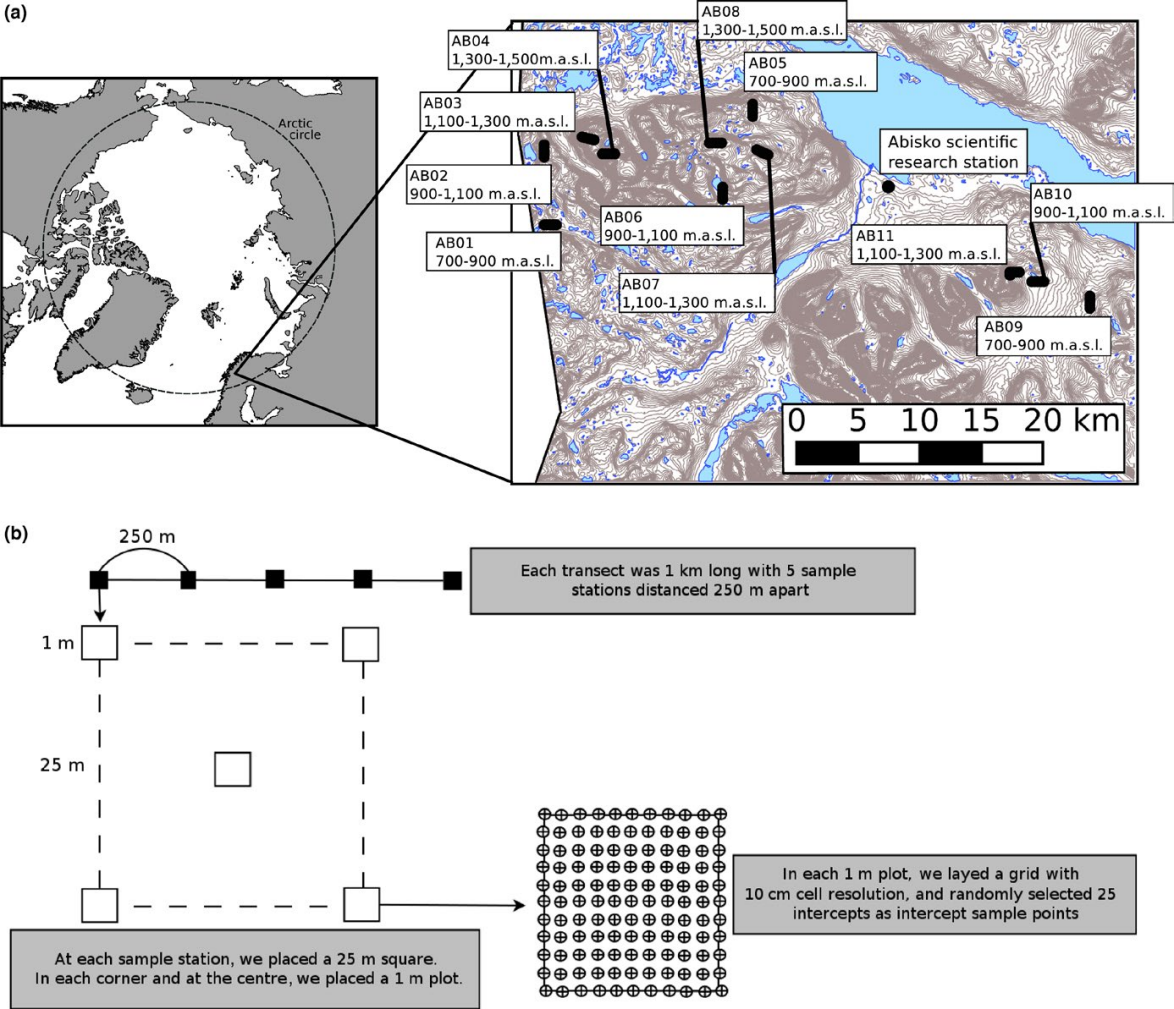
A map of the study area including the transect locations and the Abisko scientific research station (a), and a schematic outline of the sampling transects (b). Time and weather constraints prevented us from sampling the highest elevation at the southeast mountain, which subsequently only contained three transects

We collected field data on mountains west and southeast of the Abisko Scientific Research Station (ANS), which is located approximately 200 km north of the Arctic circle (68°21′N, 18°49′E, Figure 1a). The area is dominated by glacial valleys and mountains up to 1,750 m above sea level (Klaus, Becher, & Klaminder, 2013), with the tree line occurring at approximately 650 m (Dahlberg, Berge, Petersson, & Vencatasawmy, 2004). Climate is varied with a maritime influence from the west and a continental influence from the east (Klaus et al., 2013). The subalpine vegetation is dominated by mountain birch. Woodlands are characterized by heath with a field layer dominated by dwarf shrubs. Herb‐rich, meadow‐type forests occur in areas of moving groundwater and are often dominated by broad‐leaf grasses and tall herbs (Callaghan, Carlsson, & Svensson, 1996). Above the tree line, vegetation consists of a mixture of diverse plant communities, including cushion plant communities characterized by *Saxifraga oppositifolia*, dwarf shrub communities consisting of *Betula nana* as well as different *Salix* and *Vaccinium* species, and heath communities mainly consisting of *Juncus, Carex,* and various grass (Poaceae) species (Callaghan et al., 1996).

The mean annual temperature at Abisko increased between 1913 and 2006, with a mean annual temperature of 0.06°C during 2006 and mean monthly temperatures ranging from −10.8°C in January to +11.7°C in July (Callaghan et al., 2010). The mean annual precipitation for the same time period was 305 mm, with monthly precipitation ranging from 11.5 mm in April to 50.7 mm in July. From around 24th May until 20th July, the time period incorporating the majority of the fieldwork, the sun does not set (Bäckman, Karlsson, & Alerstam, 2015).

### 2.2 Field data collection

Field data were collected during July 2016 along 11 pre‐identified 1 km transect lines distributed across 3 mountains (Figure 1a). Each transect was placed within one of four elevation bands, 700–900 m, 900–1,100 m, 1,100–1,300 m and >1,300 m. Each mountain had one transect within each elevation band. However, due to time and weather constraints, we could not sample the highest elevation transect on one mountain. The transects were placed so they had minimal altitude variation among the sample stations (mean *SD* for all transects 15.6 m, range 6.3–28.8 m). In addition, we placed the transects so that they avoided lakes and rivers to minimize logistic difficulties, and to not be influenced by footpaths, trails, or other human infrastructure.

The 1 km long transects consisted of five sample stations spaced 250 m apart. At each of the five stations, a 25 × 25 m square was placed. At each of the four corners and the center point of the square, we placed a 1 m^2^ sample plot (Figure 1b). A visual estimation of the total percentage of vegetation cover was noted for each plot, and we identified all vascular plants to species level if possible. We used a modified version of the ITEX (International Tundra Experiment) community baseline monitoring protocol to quantify the relative abundance of species within our plots (Walker, 1996). Within each plot, we placed a grid of 100 10 × 10 cm cells from which 20 random cell intersections were selected as sample points. The 20 intersections were combined with each corner and the center of each grid for a total of 25 sample points for each plot, amounting to 125 for each station and 625 for each 1 km transect. At each intersection, we placed an upright stick and counted any plant that was touching it.

We identified plants to species level in the field if possible. However, when field identification was not possible, we brought representative specimens back to the station to compare with a reference collection. Despite these efforts, 6.6% of the individual plants could only be identified to genus (99 out of 1,491 taxonomic identifications). These are outlined in detail in Supporting Information Table S1 and were distributed across all three mountains and occurred in 10 out of the 11 transects.

### 2.3 Data analysis

We used the visually estimated percentage cover in each 1 m^2^ plot as an indicator of vascular plant cover and the number of identified species in each 1 m^2^ plot as an indicator of vascular plant species richness. We used the number of times each species was observed in the cell intersections as an indicator of the relative community composition of vascular plant species. We pooled all intercepts for a whole station, that is, for five 1 m^2^ plots, since we got too few species intercepts in individual plots to accurately quantify the relative abundance of different species. We used a scaled Shannon diversity index as an estimate of species evenness within each station (Tóthmérész, 1998). This index provides a measure of evenness in relative species abundances, and has been regarded as appropriate for biodiversity quantifications (Jost, 2006). For ease of interpretation, we scaled the index so that a value of 1 represented complete evenness and a value of 0 a community with only one species.

In accordance with Anderson, Ellingsen, and McArdle (2006), we used the average dissimilarity between each plot and relevant group centroids (see below) as an index of beta diversity. This measure has been regarded as appropriate to measure beta diversity across and along gradients (Anderson et al., 2006). We calculated the group centroids in several steps and for three spatial scales. First, we used a binary Bray–Curtis dissimilarity to create a pairwise distance matrix describing the difference in binary community compositions among stations. We then used this distance matrix to calculate the differences between each plot and three centroids representing three spatial scales, (a) the sample station, representing beta diversity on a very local (25 × 25 m) scale, (b) transect, representing beta diversity on a local (1 km) scale, and (c) among all plots within the same altitude band, representing beta diversity on the same altitude at a landscape scale. To enable the use of a dissimilarity metric that does not satisfy triangle inequality, the pairwise distances were first converted into principal coordinate space prior to calculation of Euclidean distances (Anderson et al., 2006).

We used mixed linear models to evaluate the effect of altitude on percentage cover of vascular plants and species evenness. In the model on cover, we used the logit transformed proportion of cover in each plot as the response variable (Warton & Hui, 2011), and in the model on species evenness we used the scaled Shannon index. We used a generalized mixed linear model with a log‐link function and a Poisson error structure to evaluate the effect of altitude on species richness. This model had the raw number of species per plot as the response variable. We used station nested in transect and mountain as a random effect structure for the models on cover and species richness and transect nested in mountain as a random effect structure for the model on species evenness. To evaluate potential nonlinearity in the relationships, we evaluated both linear and quadratic relationships between each response variable and altitude. We used likelihood ratio tests to evaluate if the increased complexity of the quadratic relationship significantly improved the model fit.

We similarly used mixed linear models to evaluate the effects of altitude and spatial scale on beta diversity. We used the Euclidean distance between each plot and the relevant centroid as response variable. We fitted two models, one using a linear relationship between beta diversity and altitude and one where we also introduced a quadratic term. In both models, we added the type of centroid (i.e., station, transect, or landscape) and the two‐way interaction between type of centroid and the altitude covariates as fixed effects. We used Tukey posthoc contrast to evaluate pairwise differences in beta diversity among the different spatial scales, and we evaluated effects of altitude within each scale using subset models. For all models, we added station nested in transect and mountain as a random effect structure.

We measured nestedness using the deterministic NODF (nestedness metric based on overlap and decreasing fill) algorithm (Almeida‐Neto, Guimaraes, Guimarães, Loyola, & Ulrich, 2008) and modularity using Barber's *Q* metric based on the BRIM (Bipartite Recursively Induced Modules) algorithm (Barber, 2007). The NODF index can range between 0 (indicating no nestedness) and 100 (indicating perfect nestedness), but is dependent on matrix fill so that appropriate interpretations rely on comparisons with appropriate null models (Almeida‐Neto et al., 2008). Barber's *Q* is a bipartite extension to Newman and Girvan's (2004) initial concept of network modularity and is similarly to the NODF index relying on null models for proper interpretation. It has been suggested as a powerful method for identifying spatial modularity in species distribution (Dallas, 2018). Both nestedness and modularity analyses were conducted on binary species presence matrices with the 1 m^2^ plots as rows and the plant species as columns. We calculated separate values of both nestedness and modularity for all transects pooled as well as for each individual mountain. For both of these scales, we calculated nestedness and modularity based on an optimal sorting of rows and columns. For nestedness, we sorted the plots based on species richness and species based on abundances (Ulrich, Almeida‐Neto, & Gotelli, 2009). For modularity, we sorted plots and species based on respective plot and species scores derived from reciprocal averaging (Hill, 1973). To enable a direct quantification of the effect of altitude on community organization, we also calculated nestedness and modularity values based on matrices where the plots were sorted based on the corresponding altitude (Dalerum et al., 2017). For modularity, we also constructed a matrix where plots were sorted by altitude within each mountain. We compared our observed values of nestedness and modularity to random expectations using 1,000 randomised matrices based on a null model algorithm that preserves species frequencies (Jonsson, 2001). We evaluated each observed value of nestedness and modularity against the null model expectations using a simple *Z*‐score conversion.

Statistical analyses were conducted in the statistical environment R version 3.4.4 for Linux (http://www.r-project.org) using the user‐contributed packages vegan (Oksanen et al., 2018), emmeans (Lenth, 2018), metacom (Dallas, 2018), and lme4 (Bates, Maechler, Bolker, & Walker, 2015).

## 3 RESULTS

**Figure 2:**
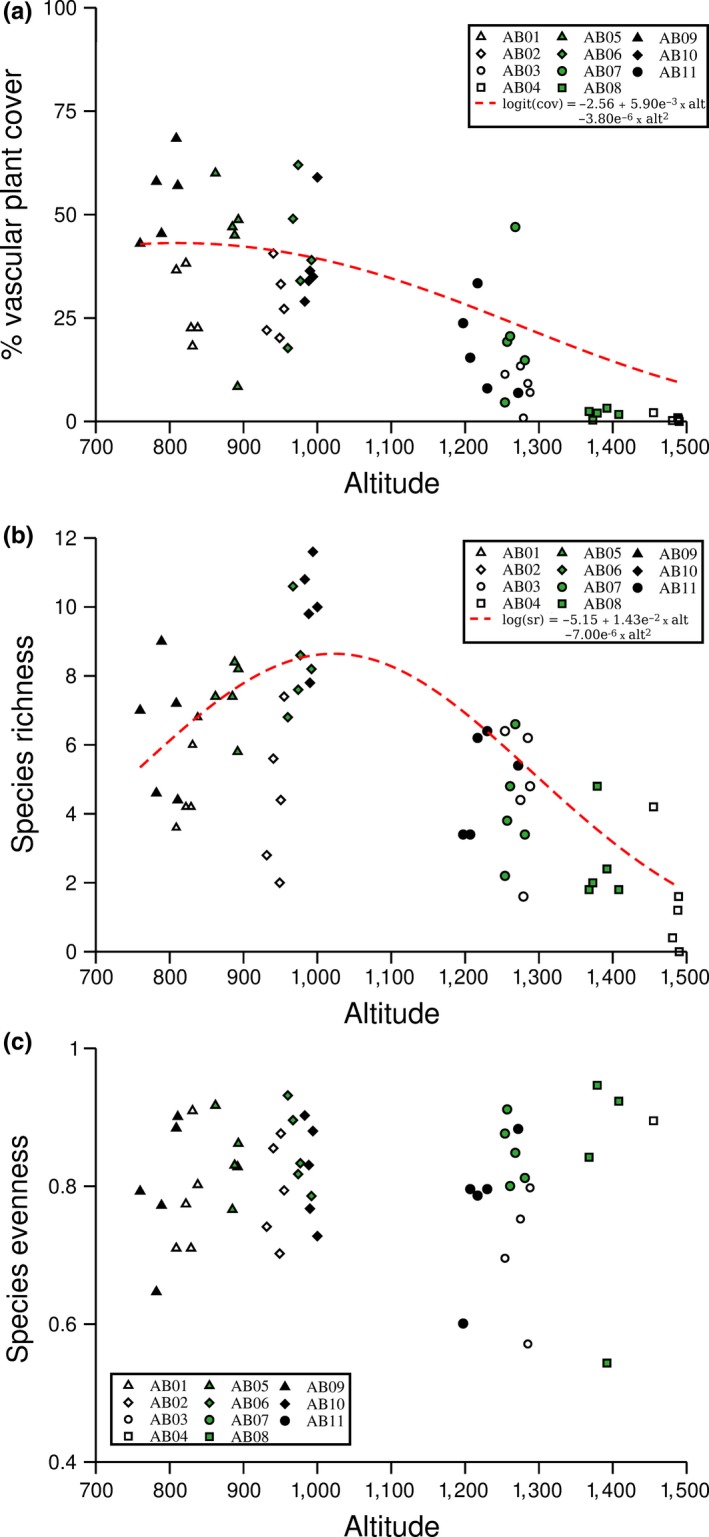
Vascular plant cover (%, a), species richness (b), and species evenness (c) in 1 m^2^ plots at varying altitudes. The plots were clustered in groups of five, representing one sample station, which were distributed across 11 transects located at different altitudes. Each point represents the average of the five 1 m^2^ plots within each station, and the red hashed lines present significant quadratic relationships between altitude and % vascular plant cover (a) and species richness (b). There were neither significant linear nor quadratic relationships between altitude and species evenness

We identified a total of 139 plant species from 68 genuses (Supporting Information Table S1). For both %, a quadratic relationship with altitude provided a significantly better fit than a linear one (cover *χ* ^2^ = 4.44, *df* = 1, *p* = 0.035; species richness: *χ* ^2^ = 13.65, *df* = 1, *p* < 0.001), with a peak in % cover at 800–900 m.a.s.l. (Figure 2a) and in species richness at approximately 1,000 m.a.s.l. (Figure 2b). The equations for both % cover (*β* = −3.80 × 10^−6^, *SE*_b_ = 1.70 × 10^−6^, *p* = 0.029) and species richness (*β* = −7.00 × 10^−6^, *SE*_b_ = 1.60 × 10^−6^, *p* < 0.001) contained significant quadratic terms, but only the equation for species richness contained a significant linear term (% cover: *β* = −5.90 × 10^−3^, *SE*_b_ = 3.74 × 10^−3^, *p* = 0.122; species richness: *β* = 1.43 × 10^−2^, *SE*_b_ = 3.49 × 10^−3^, *p* < 0.001). There was no significant linear relationship between Shannon evenness and altitude (*β* = −1.26 × 10^−5^, *SE*_b_ = 6.63 × 10^−5^, *p* = 0.854), and a quadratic term did not provide a significantly better fit (*χ* ^2^ = 0.01, *df* = 1, *p* = 0.938, Figure 2c).

**Figure 3:**
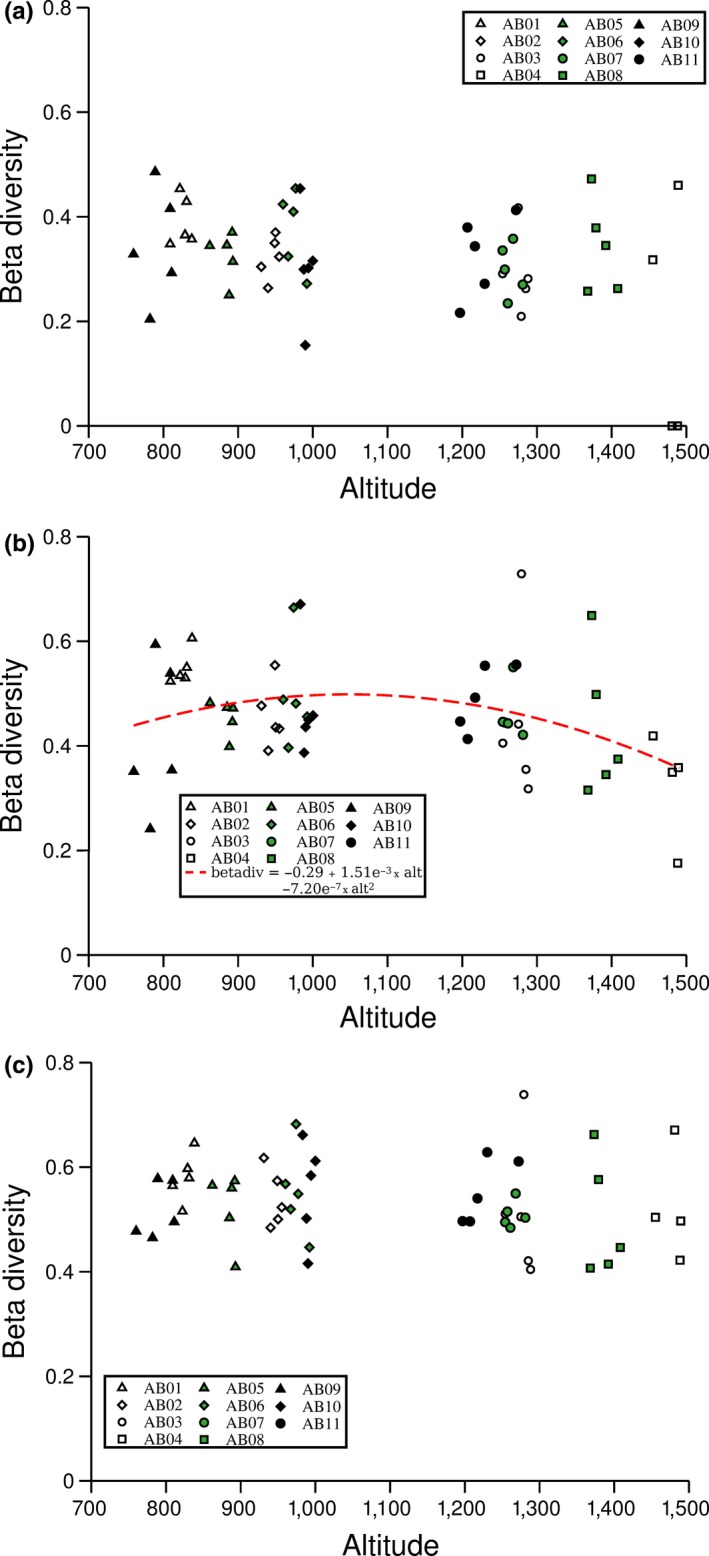
Vascular plant beta diversity at a local 25 m scale (a), at a 1 km transect scale (b) and at a 20–30 km landscape scale (c) at different altitudes, as well as a significant quadratic relationship between altitude and beta diversity at the transect scale (b). There were no significant relationships between beta diversity and altitude for either the local or the landscape scales. Beta diversity was estimated as the Euclidean distance from 1 m^2^ plots to the multidimensional centroids of all plots within a sample station (i.e., five plots clustered within a 25 × 25 m^2^square, a), of all plots along a transect of five sample stations (distributed across 1 km on a common altitude, b) and of all plots within each of four (700–900 m.a.s.l, 900–1,100 m.a.s.l., 1,100–1,300 m.a.s.l. and over 1,300 m.a.s.l.) altitude band across the whole Abisko area (approximately 40 km × 40 km) (c)

The quadratic relationship provided a significantly better fit than the linear one between beta diversity and altitude (*χ* ^2^ = 9.06, *df* = 1, *p* = 0.029). However, there was a significant interaction between the quadratic term of altitude and spatial scale (*F* = 9.06, *df* = 2,696, *p* = 0.027), with a significant quadratic term for the transect scale (*β* = −7.20 × 10^−7^, *SE*_b_ = 3.00 × 10^−7^, *p* = 0.046, Figure 3b) but not for the station (*β* = 2.00 × 10^−7^, *SE*_b_ = 3.00 × 10^−7^, *p* = 0.490, Figure 3a) or the landscape scales (*β* = 2.30 × 10^−7^, *SE*_b_ = 3.00 × 10^−7^, *p* = 0.398, Figure 3c). The linear terms were not significant for any of the three spatial scales (station: *β* = −5.41 × 10^−4^, *SE*_b_ = 6.64 × 10^−5^, *p* = 0.420; transect: *β* = 1.51 × 10^−3^, *SE*_b_ = 7.75 × 10^−4^, *p* = 0.058; landscape: *β* = 4.64 × 10^−4^, *SE*_b_ = 6.08 × 10^−4^, *p* = 0.450). Across all altitudes, vascular plant beta diversity at the station scale was smaller than for both transect (mean difference = 0.17, *t* = 9.21, *df* = 696, *p* < 0.001) and landscape (mean difference = 0.22, *t* = 11.99, *df* = 696, *p* < 0.001) scales, and beta diversity at the transect scale was smaller than for the landscape scale (mean difference = 0.05, *t* = 2.77, *df* = 696, *p* = 0.015).

**Figure 4:**
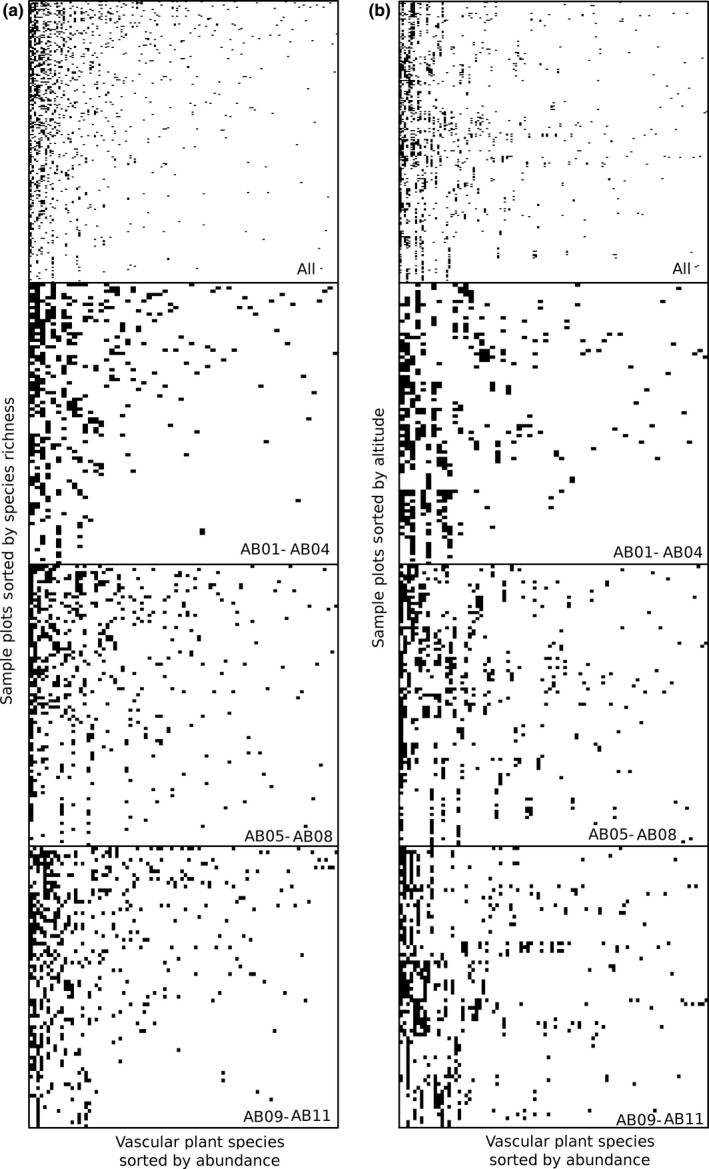
Image representations of binary matrices representing presence or absence of plant species (columns) within each 1 m^2^ sample plot (rows) for all transects pooled as well as for each of three sampled mountains separately, sorting plant species based on their abundance and sorting sites for optimal nestedness based on species richness (a), and on altitude (b)

**Figure 5:**
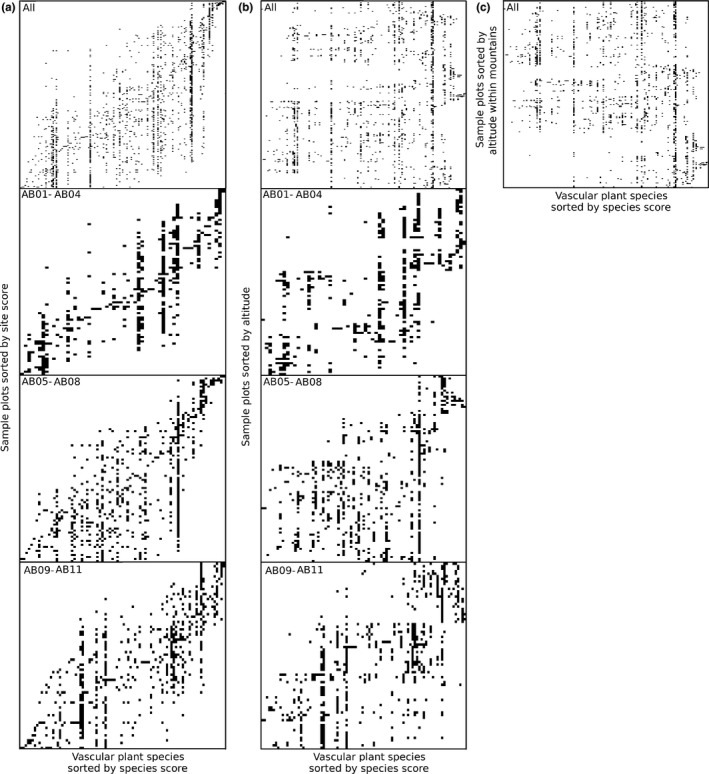
Image representations of binary matrices representing presence or absence of plant species (columns) within each 1 m^2^ sample plot (rows) for all transects pooled as well as for each of three sampled mountains separately, sorting plant species based on their reciprocal averaging scores and sorting sites based on reciprocal averaging scores for optimal modularity (a), altitude (b) and altitude within each mountain (c)

Community composition consistently showed a more nested pattern than random expectations (Table 1), both for all mountains pooled as well as for each mountain separately (Figure 4a). However, this nestedness did not follow the altitude gradient (Figure 4b), with the altitude sorted matrices both for the full data set and for each separate mountain exhibiting less nestedness than expected by the null model (Table 1). There was also evidence for a modular pattern of community composition (Table 1), both for all areas pooled and for each separate mountain (Figure 5a). Contrarily to nestedness, the modular pattern was still evident when sorting plots based on altitude, with both the altitude sorted matrices and the full matrix with altitude sorted within each mountain (Table 1). The modular pattern along altitude was particularly evident for individual mountains (Figure 5b), and less so for the matrix sorted by altitude within mountains (Figure 5c).

**Table 1:** Observed and expected values of the NODF index of spatial nestedness and Barber's *Q* index of spatial modularity for optimally sorted matrices, where the plots were sorted by species richness for nestedness and by the rank order derived from reciprocal averaging for modularity, as well as matrices where plots were sorted by altitude and by altitude within mountains, as well as associated *Z* statistics

Transects	Sorting of plots	Nestedness (NODF)				Modularity (*Q*)			
		Obs	Exp	*Z*	*p*	Obs	Exp	*Z*	*p*
All	Optimal	20.10	17.27	7.64	<0.001	0.33	0.22	10.7	<0.001
	Altitude	12.66	17.27	−2.43	<0.001	0.33	0.22	9.92	<0.001
	Altitude within mountain					0.33	0.22	9.92	<0.001
AB01–AB04	Optimal	24.30	21.70	3.09	0.001	0.32	0.28	6.23	<0.001
	Altitude	16.11	21.70	−6.65	<0.001	0.32	0.28	6.23	<0.001
AB05–AB08	Optimal	20.47	18.49	3.18	0.001	0.38	0.26	10.6	<0.001
	Altitude	16.02	18.49	−3.94	<0.001	0.38	0.26	9.05	<0.001
AB09–AB11	Optimal	19.00	16.63	5.27	<0.001	0.38	0.26	9.51	<0.001
	Altitude	14.28	16.64	−5.21	<0.001	0.38	0.26	9.37	<0.001

## 4 DISCUSSION

Both vascular plant cover and species richness declined with altitude, but contrary to some observations (Ah‐Peng et al., 2012; Larjavaara & Muller‐Landau, 2012), our study did not suggest that such declines were monotonic. Instead, our study agrees with previous findings of unimodal relationships between altitude and vascular plant species richness (Bruun et al., 2006; Grytnes, 2003; Rahbek, 1995). Such relationships have been suggested as the consequence of productivity associated competitive exclusions at lower altitudes (following from Rosenzweig, 1971) and productivity‐related dilutions of regional species pools at higher altitudes (Huston, 1999). Despite the observed declines in vegetation cover and species richness, we did not observe an associated decline in species evenness, so that that not one species appears to have become dominant as species communities became diluted at higher altitudes. Although this result partly could have been caused by a declining species pool at higher altitudes (Dalerum et al., 2017), our observations support suggestions of strong abiotic regulation of plant diversity at higher altitudes (Sang, 2009), coupled with relaxed competition (Bruun et al., 2006). Since global warming is likely to release some of the abiotic constraints (Parmesan & Yohe, 2003), our results highlight that high altitude plant communities are likely to change dramatically under credible climate change scenarios. Such shifts have already been observed (Gottfried et al., 2012; Pauli et al., 2012), but are likely to be unpredictable due strong interactions between biotic and abiotic regulation of plant communities (Wilson & Nilsson, 2009).

We found a predicted scale dependency of beta diversity, with the patch scale (sample station) having lower beta diversity than the intermediate (transect) or landscape scales, and the intermediate scale having lower beta diversity than the landscape scale. These results contradict observations pointing to a strong importance of local characteristics and processes for shaping biodiversity patterns in plants (Marini, Scotton, Klimek, & Pecile, 2008) and invertebrates (Dalerum et al., 2019, 2017), and instead suggest that broad‐scale environmental variation may have been more important for the spatial heterogeneity of plant communities than patch level characteristics. Although local characteristics such as snow cover (When, Lundemo, & Holten, 2014), soil and topographic properties (Marini, Scotton, Klimek, Isselstein, & Pecile, 2007), and geological substrate (Trigas, Tsiftsis, Tsiripidis, & Iatrou, 2012) all influence local plant communities, the magnitude of effects of local conditions may be dictated by constraints set by landscape characteristics. Hence, it is important to recognize that factors at various scales are not acting in isolation, but have synergetic effects which may include the interactions with other plants (Godsoe, Jankowski, Holt, & Gravel, 2017; Grassein, Lavorel, & Till‐Bottraud, 2014; Tilman, 2004), symbionts (Gardes & Dahlberg, 1996) and with herbivores (Austrheim & Eriksson, 2001).

We also observed scale dependence in the relationship between altitude and beta diversity, with a unimodal relationship between altitude and beta diversity only for the intermediate (transect) scale. This relationship corresponded to the relationship between altitude and species richness, with an increased spatial heterogeneity of plant communities at lower altitudes and a spatial homogenization at higher ones. Hence, at this spatial scale, spatial heterogeneity appears to, at least partly, be maintained by the available species pool and subsequently by local variations in species deletions from this available pool. However, the lack of an altitudinal change in beta diversity at either patch or landscape scales suggests that the processes that generate spatial heterogeneity across altitude occur at spatial scales larger than our 25 × 25 m sample stations, and that there was no general factor homogenizing plant communities across our three mountains. This latter observation contradicts findings of spatial homogenization at high altitudes, caused by upward shifts in ubiquitous alpine species altitudes (Jurasisnski & Kreyling, 2007; Odland, Hoitomt, & Olsen, 2010).

We observed both nested and modular community structures, but only the modular structures appeared to correspond to shifts in altitude. This latter result highlights that the main driver behind altitude variation in plant communities appear to have been species turnover, with species assemblages specific for each section along the altitude gradient. Such structuring could be caused by both ecologic constrains limiting individual species occurrences, phylogenetic constraints associated with adaptations to local conditions, or a combination of the two (Graham et al., 2014). In contrast to modularity, the composition of plant species communities appears to have been nested according to factors not associated with altitude, such as for instance soil characteristics, hydrology, and light regime (Körner, 2003). This combination of nested and modular structures highlights the need for further studies evaluating how altitude effects are influenced by spatial scales as well as context‐dependent processes (Callaghan et al., 2013).

We acknowledge some caveats with this study. First, our imperfect taxonomic identification, where we did not have all plants identified to species level in all the cases, could have influenced both plant species richness and estimates of species evenness and beta diversity. However, only a small portion of the identified plants were not resolved to species level, and these were distributed across all sampled mountains and occurred in almost all transects. Hence, we suggest that our imperfect taxonomic identification may have had limited effects on the identified community patterns. Second, the study had a limited number of sample sites across only three elevation gradients. The geological structure of the Abisko area is complex, with a great spatial variation in the locally prevailing bedrock and in the geological substrate (Kozlowska & Raczkowska, 2002). This variation has likely influenced plant communities and generated hotspots of species richness at high altitudes, including unique endemic flora (Trigas et al., 2012). Although we did not stratify our sampling by geological substrate, we took great care in not biasing the transects with regards to geology. Subsequently, we argue that our results are not biased and that any spatial variation caused by substrate variation only has influenced the precision in our estimates of altitude effects on vascular plant communities. Finally, our analyses only contain taxonomic diversity, which has been suggested as a crude proxy for more functional and evolutionary relevant measurements of species variations (Dalerum, 2013).

To conclude, we found unimodal relationships between altitude and vascular plant cover and plant species richness, but altitude had little effect on plant species evenness. A unimodal relationship between altitude and beta diversity at the transect scale suggested a spatial homogenization at higher altitudes, but a lack of relationships with altitude for either the station or landscape scales suggests that altitude effects on plant spatial heterogeneity occurred on scales larger than individual patches but were not effective across the whole landscape. We found both nested and modular patterns of species distributions, but only the modular patterns corresponded to the altitude gradient. Overall, our observations supported biotic regulations of plant communities at high altitudes in this subarctic tundra, but strong scale dependencies and inconsistent magnitude of the effects of altitude on different components of the taxonomic diversity of vascular plants highlight the need for further studies evaluating how different drivers influence biodiversity in high altitude and high latitude environments, as well as studies identifying scale and context dependencies in the effects of such drivers.

## CONFLICT OF INTERESTS

None declared.

## AUTHOR CONTRIBUTIONS

LN conducted field sampling, assisted with data analyses, and wrote major parts of the manuscript, JM contributed with data interpretation and manuscript writing, SF conducted fieldwork, organized data for analyses and assisted with data interpretation, AA and LD assisted with sampling design, and FD conceptualized the study, assisted with field sampling, designed and conducted the data analyses and wrote parts of the manuscript.

## Data Availability

Data on vegetation cover, species presences, Shannon evenness, and centroid distances used for estimation of beta diversity, as well as a binary presence–absence matrix, are available from datadryad (https://datadryad.org/, https://doi.org/10.5061/dryad.t7874hd/1).

## References

[ece35081-bib-0001] Ah‐Peng, C. , Wilding, N. , Kluge, J. , Descamps‐Julien, B. , Bardat, J. , Chuah‐Petiot, M. , … Hedderson, T. A. J. (2012). Bryophyte diversity and range size distribution along two altitudinal gradients: Continent vs. island. Acta Oecologica‐International Journal of Ecology, 42, 58–65. 10.1016/j.actao.2012.04.010

[ece35081-bib-0002] Almeida‐Neto, M. , Guimaraes, P. , Guimarães, P. R. , Loyola, R. D. , & Ulrich, W. (2008). A consistent metric for nestedness analysis in ecological systems: Reconciling concept and measurement. Oikos, 117, 1227–1239. 10.1111/j.0030-1299.2008.16644.x

[ece35081-bib-0003] Anasimov, O. A. , Vaughan, D. G. , Callaghan, T. V. , Furgal, C. , Marchant, H. , Prowse, T. D. , … Walsh, J. E. (2007). Polar regions (Arctic and Antarctic) In Parry M. L., Canziani O. F., Palutikof J. P., van der Linden P. J., & Hanson C. E. (Eds.), Climate change 2007: Impacts, adaptation and vulnerability (pp. 653–686). Cambridge, UK: Cambridge University Press.

[ece35081-bib-0004] Anderson, M. J. , Ellingsen, K. E. , & McArdle, B. H. (2006). Multivariate dispersion as a measure of beta diversity. Ecology Letters, 9, 683–693. 10.1111/j.1461-0248.2006.00926.x 16706913

[ece35081-bib-0005] Austrheim, G. , & Eriksson, O. (2001). Plant species diversity and grazing in the Scandinavian mountains – patterns and processes at different spatial scales. Ecography, 24, 683–695. 10.1034/j.1600-0587.2001.240607.x

[ece35081-bib-0006] Barber, M. J. (2007). Modularity and community detection in bipartite networks. Physical Review E, 76, 066102 10.1103/PhysRevE.76.066102 18233893

[ece35081-bib-0007] Baselga, A. (2010). Partitioning the turnover and nestedness components of betadiversity. Global Ecology and Biogeography, 19, 134–143. 10.1111/j.1466-8238.2009.00490.x

[ece35081-bib-0008] Bastolla, U. , Fortuna, M. A. , Pascual‐Garcia, A. , Ferrera, A. , Luque, B. , & Bascompte, J. (2009). The architecture of mutualistic networks minimizes competition and increases biodiversity. Nature, 458, 1018–1020. 10.1038/nature07950 19396144

[ece35081-bib-0009] Bates, D. , Maechler, M. , Bolker, B. , & Walker, S. (2015). Fitting linear mixed‐effects models using lme4. Journal of Statistical Software, 67, 4783–48. 10.18637/jss.v067.i01

[ece35081-bib-0010] Beniston, M. (2003). Climatic change in mountain regions: A review of possible impacts. Climate Change, 59, 5–31. 10.1023/A:1024458411589

[ece35081-bib-0011] Billings, W. D. , & Mooney, H. A. (1968). The ecology of arctic and alpine plants. Biological Reviews, 43, 481–529. 10.1111/j.1469-185X.1968.tb00968.x

[ece35081-bib-0012] Bliss, L. C. (1956). A comparison of plant development in microenvironments of arctic and alpine tundras. Ecological Monographs, 26, 303–337. 10.2307/1948544

[ece35081-bib-0013] Bliss, L. C. (1971). Arctic and alpine life cycles. Annual Review of Ecology and Systematics, 2, 405–438. 10.1146/annurev.es.02.110171.002201

[ece35081-bib-0014] Brown, J. H. (2001). Mammals on mountainsides: Elevational patterns of diversity. Global Ecology and Biogeography, 10, 101–109. 10.1046/j.1466-822x.2001.00228.x

[ece35081-bib-0015] Bruun, H. H. , Moen, J. , Virtanen, R. , Grytnes, J. A. , Oksanen, L. , & Angerbjörn, A. (2006). Effects of altitude and topography on species richness of vascular plants, bryophytes and lichens in alpine communities. Journal of Vegetation Science, 17, 37–46. 10.1111/j.1654-1103.2006.tb02421.x

[ece35081-bib-0016] Callaghan, T. V. , Bergholm, F. , Christensen, T. , Jonasson, C. , Kokfelt, U. , & Johansson, M. (2010). A new climate era in the sub‐arctic? Accelerating climate changes and multiple impacts. Geophysical Research Letters, 37, L14705 10.1029/2009GL042064

[ece35081-bib-0017] Callaghan, T. V. , Carlsson, B. A. , & Svensson, B. M. (1996). Some apparently paradoxical aspects of the life cycles, demography and population dynamics of planta from the subarctic Abisko area. Ecological Bulletins, 45, 133–143.

[ece35081-bib-0018] Callaghan, T. V. , Jonasson, C. , Thierfelder, T. , Yang, Z. L. , Hedenas, H. , Johansson, M. , … Sloan, V. L. (2013). Ecosystem change and stability over multiple decades in the Swedish subarctic: Complex processes and multiple drivers. Philosophical Transactions of the Royal Society B – Biological Sciences, 368, 20120488 10.1098/rstb.2012.0488 PMC3720059 23836792

[ece35081-bib-0019] Chapin III, F. S. , & Körner, C. (1995). Patterns, causes, changes, and consequences of biodiversity in arctic and alpine ecosystems In Chapin F. S. III, & Körner C. (Eds.), Arctic and alpine biodiversity: Patterns, causes and ecosystem consequences (pp. 313–320). Berlin, Germany: Springer Verlaag.

[ece35081-bib-0020] Chesson, P. (2000). Mechanisms of maintenance of species diversity. Annual Review in Ecology and Systematics, 31, 343–366. 10.1146/annurev.ecolsys.31.1.343

[ece35081-bib-0021] Colwell, R. K. , & Lees, D. C. (2000). The mid‐domain effect: Geometric constraints on the geography of species richness. Trends in Ecology and Evolution, 15, 70–76. 10.1016/S0169-5347(99)01767-X 10652559

[ece35081-bib-0022] Currie, D. J. (1991). Energy and large‐scale patterns of animal – and plant – species richness. American Naturalist, 137, 27–49. 10.1086/285144

[ece35081-bib-0023] Currie, D. J. , Mittelbach, G. G. , Cornell, H. W. , Field, R. , Guegan, J. F. , Hawkins, B. A. , … Turner, J. R. G. (2004). Predictions and tests of climate‐based hypotheses of broad‐scale variation in taxonomic richness. Ecology Letters, 7, 1121–1134. 10.1111/j.1461-0248.2004.00671.x

[ece35081-bib-0024] Dahlberg, U. , Berge, T. W. , Petersson, H. , & Vencatasawmy, C. P. (2004). Modelling biomass and leaf area index in a aub‐Arctic Scandinavian mountain area. Scandinavian Journal of Forest Research, 19, 60–71. 10.1080/02827580310019266

[ece35081-bib-0025] Dalerum, F. (2012). Descriptive versus explanatory hypotheses in evolutionary research; A potentially concerning bias exemplified by research into the evolution of social organisations among carnivores. Ethology, Ecology, and Evolution, 24, 97–103. 10.1080/03949370.2011.582043

[ece35081-bib-0026] Dalerum, F. (2013). Phylogenetic and functional diversity in large carnivore assemblages. Proceedings of the Royal Society B: Biological Sciences, 280, 20130049 10.1098/rspb.2013.0049 PMC3652454 23576787

[ece35081-bib-0027] Dalerum, F. , de Vries, J. L. , Pirk, C. W. W. , & Cameron, E. Z. (2017). Spatial and temporal dimensions to the taxonomic diversity of arthropods in an arid grassland savannah. Journal of Arid Environments, 144, 21–30. 10.1016/j.jaridenv.2017.04.002

[ece35081-bib-0028] Dalerum, F. , Retief, T. , Havemann, C. P. , Chimimba, C. T. , & Van Rensburg, B. J. (2019). The influence of distance to perrenial surface water on ant diversity in Mopane Woodlands, northern Botswana. Ecology and Evolution, 9, 154–165. 10.1002/ece3.4692 30680103 PMC6342134

[ece35081-bib-0029] Dallas, T. (2018). metacom: Analysis of the 'Elements of Metacommunity Structure’. Retrieved from https://CRAN.R-project.org/package=metacom

[ece35081-bib-0030] Emerson, B. C. , & Gillespie, R. G. (2008). Phylogenetic analysis of community assembly and structure over space and time. Trends in Ecology & Evolution, 23, 619–630. 10.1016/j.tree.2008.07.005 18823678

[ece35081-bib-0031] Galeano, J. , Pastor, J. M. , & Iriondo, J. M. (2009). Weighted‐Interaction Nestedness Estimator (WINE): A new estimator to calculate over frequency matrices. Environmental Modelling Software, 24, 1342–1346. 10.1016/j.envsoft.2009.05.014

[ece35081-bib-0032] Gardes, N. , & Dahlberg, A. (1996). Mycorrhizal diversity in arctic and alpine tundra: An open question. New Phytologist, 133, 147–157. 10.1111/j.1469-8137.1996.tb04350.x

[ece35081-bib-0033] Gaston, K. J. (2000). Global patterns in biodiversity. Nature, 405, 220–227. 10.1038/35012228 10821282

[ece35081-bib-0034] Gaston, K. J. , & Blackburn, T. M. (2000). Patterns and processes in macroecology. Oxford, UK: Blackwell Science.

[ece35081-bib-0035] Godsoe, W. , Jankowski, J. , Holt, R. D. , & Gravel, D. (2017). Integrating biogeography with contemporary niche theory. Trends in Ecology and Evolution, 32, 488–499. 10.1016/j.tree.2017.03.008 28477957

[ece35081-bib-0036] Gottfried, M. , Pauli, H. , Futschik, A. , Akhalkatsi, M. , Barančok, P. , Alonso, J. L. B. , … Grabherr, G. (2012). Continent‐wide response of mountain vegetation to climate change. Nature Climate Change, 2, 111–114. 10.1038/nclimate1329

[ece35081-bib-0037] Grabherr, G. , Gottfried, M. , & Pauli, H. (1994). Climate effects on mountain plants. Nature, 369, 448 10.1038/369448a0 23320303

[ece35081-bib-0038] Graham, C. H. , Carnaval, A. C. , Cadena, C. D. , Zamudio, K. R. , Roberts, T. E. , Parra, J. L. , … Sanders, N. J. (2014). The origin and maintenance of montane diversity: Integrating evolutionary and ecological processes. Ecography, 37, 711–719. 10.1111/ecog.00578

[ece35081-bib-0039] Grassein, F. , Lavorel, S. , & Till‐Bottraud, I. (2014). The importance of biotic interactions and local adaptation for plant response to environmental changes: Field evidence along an elevational gradient. Global Change Biology, 20, 1452–1460. 10.1111/gcb.12445 24306968

[ece35081-bib-0040] Grytnes, J. A. (2003). Species‐richness patterns of vascular plants along seven altitudinal transects in Norway. Ecography, 29, 291–300. 10.1034/j.1600-0587.2003.03358.x

[ece35081-bib-0041] Hawkins, B. A. , Field, R. , Cornell, H. V. , Currie, D. J. , Guégan, J. F. , Kaufman, D. M. , … Turner, J. R. G. (2003). Energy, water, and broad‐scale geographic patterns of species richness. Ecology, 84, 3105–3117. 10.1890/03-8006

[ece35081-bib-0042] Hill, M. O. (1973). Reciprocal averaging: An eigenvector method of ordination. Journal of Ecology, 61, 237–249. 10.2307/2258931

[ece35081-bib-0043] Huston, M. A. (1999). Local processes and regional patterns: Appropriate scales for understanding variation in the diversity of plants and animals. Oikos, 86, 393–401. 10.2307/3546645

[ece35081-bib-0044] Jonsson, B. G. (2001). A null model for randomization tests of nestedness in species assemblages. Oecologia, 127, 309–313. 10.1007/s004420000601 28547100

[ece35081-bib-0045] Jost, L. (2006). Entropy and diversity. Oikos, 113, 363–375 10.1111/j.2006.0030-1299.14714.x.

[ece35081-bib-0046] Jurasisnski, J. , & Kreyling, J. (2007). Upward shift of alpine plants increases floristic similarity of mountain summits. Journal of Vegetation Science, 18, 711–718. 10.1111/j.1654-1103.2007.tb02585.x

[ece35081-bib-0047] Klaus, M. , Becher, M. , & Klaminder, J. (2013). Cryogenic soil activity along bioclimatic gradients in northern Sweden: lnsights from eight different proxies. Permafrost and Periglacial Processes, 24, 210–223. 10.1002/ppp.1778

[ece35081-bib-0048] Körner, C. (2003). Alpine plant life: Functional plant ecology of high mountain ecosystems. Heidelberg, Germany: Springer.

[ece35081-bib-0049] Kozlowska, A. , & Raczkowska, Z. (2002). Vegetation as a tool in the characterisation of geomorphological forms and processes: An example from the Abisko Mountains. Geografiska Annaler: Series A, Physical Geography, 84, 233–244. 10.1111/j.0435-3676.2002.00178.x

[ece35081-bib-0050] Larjavaara, M. , & Muller‐Landau, H. C. (2012). Temperature explains global variation in biomass among humid old‐growth forests. Global Ecology and Biogeography, 21, 998–1006. 10.1111/j.1466-8238.2011.00740.x

[ece35081-bib-0051] Leibold, M. A. , Holyoak, M. , Mouquet, N. , Amarasekare, P. , Chase, J. M. , Hoopes, M. F. , … Gonzalez, A. (2004). The metacommunity concept: A framework for multi‐scale community ecology. Ecology Letters, 7, 601–613. 10.1111/j.1461-0248.2004.00608.x

[ece35081-bib-0052] Lenth, R. (2018). Emmeans: Estimated marginal means. Retrieved from https://CRAN.R-project.org/package=emmeans

[ece35081-bib-0053] Lomolino, M. V. (2001). Elevation gradients of species‐density, historical and prospective views. Global Ecology and Biogeography, 10, 3–4795. 10.1046/j.1466-822x.2001.00229.x

[ece35081-bib-0054] MacArthur, R. H. (1972). Geographical ecology: Patterns in the distributions of Species. New York, NY: Harper & Row.

[ece35081-bib-0055] Marini, L. , Scotton, M. , Klimek, S. , Isselstein, J. , & Pecile, A. (2007). Effects of local factors on plant species richness and composition of Alpine meadows. Agriculture, Ecosystems & Environment, 119, 281–288. 10.1016/j.agee.2006.07.015

[ece35081-bib-0056] Marini, L. , Scotton, M. , Klimek, S. , & Pecile, A. (2008). Patterns of plant species richness in Alpine hay meadows: Local vs landscape controls. Basic and Applied Ecology, 9, 365–372. 10.1016/j.baae.2007.06.011

[ece35081-bib-0057] Newman, M. E. J. , & Girvan, M. (2004). Finding and evaluating community structure in networks. Physical Review E, 69, 026113 10.1103/PhysRevE.69.026113 14995526

[ece35081-bib-0058] Nilsson, C. , Bäckman, J. , Karlsson, H. , & Alerstam, T. (2015). Timing of nocturnal passerine migration in Arctic light conditions. Polar Biology, 38, 1453–1459. 10.1007/s00300-015-1708-x

[ece35081-bib-0059] Odland, A. , Hoitomt, T. , & Olsen, S. L. (2010). Increasing vascular plant richness on 13 high mountain summits in southern Norway since the early 1970s. Arctic, Antarctic and Alpine Research, 42, 458–470. 10.1657/1938-4246-42.4.458

[ece35081-bib-0060] Oksanen, J. , Blanchet, F. G. , Friendly, M. , Kindt, R. , Legendre, P. , McGlinn, D. , … Wagner, H. (2018). vegan: Community ecology package. Retrieved from https://CRAN.R-project.org/package=vegan

[ece35081-bib-0061] Olesen, J. M. , Bascompte, J. , Dupont, Y. L. , & Jordano, P. (2007). The modularity of pollination networks. Proceedings of the National Academy of Science, 104, 19891–19896. 10.1073/pnas.0706375104 PMC2148393 18056808

[ece35081-bib-0062] Parmesan, C. , & Yohe, G. (2003). A globally coherent fingerprint of climate change impacts across natural systems. Nature, 421, 37–42. 10.1038/nature01286 12511946

[ece35081-bib-0063] Patterson, B. D. , & Atmar, W. (1986). Nested subsets and the structure of insular mammalian faunas and archipelagoes. Biological Journal of the Linnean Society, 28, 65–82. 10.1111/j.1095-8312.1986.tb01749.x

[ece35081-bib-0064] Pauli, H. , Gottfried, M. , Dullinger, S. , Abdaladze, O. , Akhalkatsi, M. , Alonso, J. L. B. , … Grabherr, G. (2012). Recent plant diversity changes on Europe's mountain summits. Science, 336, 353–355. 10.1126/science.1219033 22517860

[ece35081-bib-0065] Rahbek, C. (1995). The elevational gradient of species richness: A uniform pattern? Ecography, 18, 200–205.

[ece35081-bib-0066] Rahbek, C. (2005). The role of spatial scale and the perception of large‐scale species‐richness patterns. Ecology Letters, 8, 224–239. 10.1111/j.1461-0248.2004.00701.x

[ece35081-bib-0067] Rosenzweig, M. L. (1971). Paradox of enrichment: Destabilization of exploitation ecosystems in ecological time. Science, 171, 385–387. 10.1126/science.171.3969.385 5538935

[ece35081-bib-0068] Sang, W. (2009). Plant diversity patterns and their relationships with soil and climatic factors along an altitudinal gradient in the middle Tianshan Mountain area, Xinjiang, China. Ecological Research, 24, 303–314. 10.1007/s11284-008-0507-z

[ece35081-bib-0069] Sørensen, T. (1941). Temperature relations and phenology of the northeast Greenland flowering plants. Meddelelser Om Grønland, 125, 4783–305.

[ece35081-bib-0070] Steinbauer, M. J. , Field, R. , Grytnes, J. A. , Trigas, P. , Ah-Peng, C. , Attorre, F. , Beierkuhnlein, C. (2016). Topography-driven isolation, speciation and a global increase of endemism with elevation. Global Ecology and Biogeography, 25, 1097–1107. 10.1111/geb.12469.

[ece35081-bib-0071] Tilman, D. (2004). Niche trade‐offs, neutrality, and community structure: A stochastic theory of resource competition, invasion, and community assembly. Proceedings of the National Academy of Science, 101, 10854–10861. 10.1073/pnas.0403458101 PMC503710 15243158

[ece35081-bib-0072] Tóthmérész, B. (1998). On the characterization of scale‐dependent diversity. Abstracta Botanica, 22, 149–156.

[ece35081-bib-0073] Trigas, P. , Tsiftsis, S. , Tsiripidis, I. , & Iatrou, G. (2012). Distribution patterns and conservation perspectives of the endemic flora of peloponnese (Greece). Folea Geobotanica, 47, 421–439. 10.1007/s12224-012-9130-4

[ece35081-bib-0074] Ulrich, W. , Almeida‐Neto, M. , & Gotelli, N. J. (2009). A consumer's guide to nestedness analyses. Oikos, 118, 3–17. 10.1111/j.1600-0706.2008.17053.x

[ece35081-bib-0075] Waide, R. B. , Willig, M. R. , Steiner, C. F. , Mittelbach, G. , Gough, L. , Dodson, S. I. , … Parmenter, R. (1999). The relationship between productivity and species richness. Annual Review of Ecology and Systematics, 30, 257–300. 10.1146/annurev.ecolsys.30.1.257

[ece35081-bib-0076] Walker, M. (1996). Community baseline measurements for ITEX studies In Molau U., & Mølgaard P. (Eds.), International Tundra experiment ITEX manual (2nd ed., pp. 39–41). Copenhagen, Denmark: Danish Polar Center.

[ece35081-bib-0077] Warton, D. I. , & Hui, F. K. (2011). The Arcsine is asinine: The analysis of proportions in ecology. Ecology, 92, 3–10. 10.1890/10-0340.1 21560670

[ece35081-bib-0078] When, S. , Lundemo, S. , & Holten, J. I. (2014). Alpine vegetation along multiple environmental gradients and possible consequences of climate change. Alpine Botany, 124, 155–164. 10.1007/s00035-014-0136-9

[ece35081-bib-0079] Whittaker, R. H. (1960). Vegetation of the Siskiyou Mountains, Oregon and California. Ecological Monographs, 2, 4783–44. 10.2307/1943563

[ece35081-bib-0080] Whittaker, R. J. , Willis, K. J. , & Field, R. (2001). Scale and species richness: Towards a general, hierarchical theory of species diversity. Journal of Biogeography, 28, 453–470. 10.1046/j.1365-2699.2001.00563.x

[ece35081-bib-0081] Williams, P. H. (1996). Mapping variations in the strengths and breadths of biogeographic transition zones using species turnover. Proceedings of the Royal Society, Series B, 263, 579–588. 10.1098/rspb.1996.0087

[ece35081-bib-0082] Willig, M. R. , Kaufman, D. M. , & Stevens, R. D. (2003). Latitudinal gradients of biodiversity: Pattern, process, scale, and synthesis. Annual Review of Ecology and Systematics, 34, 273–309. 10.1146/annurev.ecolsys.34.012103.144032

[ece35081-bib-0083] Wilson, S. D. , & Nilsson, C. (2009). Arctic alpine vegetation change over 20 years. Global Change Biology, 15, 1676–1674. 10.1111/j.1365-2486.2009.01896.x

[ece35081-bib-0084] Wilson, W. (1996). An analysis of plant growth and its control in the Arctic environtments. Annals of Botany, 30, 383–402.

[ece35081-bib-0085] Wright, D. H. , Patterson, B. D. , Mikkelson, G. M. , Cutler, A. , & Atmar, W. (1998). A comparative analysis of nested subset patterns of species composition. Oecologia, 113, 4783–20. 10.1007/s004420050348 28307284

