# Supplementary material for: Altitude effects on spatial components of vascular plant diversity in a subarctic mountain tundra
Source: Ecol Evol. 2019 Mar 22;9(8):4783–95. doi: 10.1002/ece3.5081 (PMC6476787; doi:10.1002/ece3.5081)
Supplement: Supplementary file 1 [file ECE3-9-4783-s001.pdf]

Supporting information for

Altitude effects on spatial components to vascular plant diversity in a sub-arctic mountain tundra

Lucy Naud[a], Johannes Måsviken[a,b], Susana Freire[c], Anders Angerbjörn[a], Love Dalén[a,b], Fredrik Dalerum[a,c,d,*]

[a] *Department of Zoology, Stockholm University, 10691 Stockholm, Sweden*

[b] *Department of Bioinformatics and Genetics, Swedish Museum of Natural History, Sweden*

[c] *Research Unit of Biodiversity (UMIB, UO-CSIC-PA), Oviedo University - Campus Mieres, 33600 Mieres, Spain*

[d] *Mammal Research Institute, Department of Zoology and Entomology, University of Pretoria, Private Bag X20, Hatfield, 0028 South Africa*

* *Correspondence: dalerumjohan@uniovi.es*

Table S1. Plant species and taxons identified during the study, as well as how many of the three mountains, transects, stations and plot each taxon were identified in. Taxonomic names follow the accepted names in The Plant List 1.1 (http://theplantlist.org)

Species/Taxon	Mountains	Transects	Stations	Plots
Antennaria alpina	2	4	4	5
Antennaria dioica	3	4	8	15
Antennaria lanata	1	1	1	1
Antennaria porsildii	1	1	1	1
Antennaria sp.	1	1	1	2
Anthoxanthum alpinum	3	3	8	19
Arenaria norvegica	1	1	3	6
Astragalus alpinus	1	1	1	4
Bartsia alpina	1	1	3	5
Betula nana	3	6	22	60
Bistorta vivipara	3	9	27	73
Calamagrostis lapponica	1	2	2	4
Calamagrostis neglecta	1	1	1	1
Calluna vulgaris	1	1	1	1
Cardamine bellidifolia	2	3	5	5
Carex atrata	2	3	8	18
Carex bigelowii	2	3	8	13
Carex fuliginosa	1	2	4	12
Carex glacialis	1	2	3	6
Carex heleonastes	1	1	1	1
Carex holostoma	1	1	1	1
Carex lachenalii	2	3	3	3
Carex nigra	1	1	1	1
Carex norvegica	1	1	1	2
Carex rotundata	1	1	3	4
Carex S1	1	1	1	1
Carex S2	2	3	9	26
Carex sp.	3	7	25	68
Cassiope hypnoides	3	11	28	71
Cassiope tetragona	3	7	17	32
Cerastium alpinum	2	2	2	2
Cerastium cerastoides	1	1	2	2
Cerastium nigrescens	1	1	1	1
Crepis paludosa	1	1	2	2
Deschampsia alpina	1	1	2	4
Deschampsia cespitosa	1	1	1	3
Diapensia lapponica	3	5	7	12
Diphasiastrum alpinum	2	4	7	8
Draba cacuminum	1	1	2	4
Draba norvegica	1	1	2	3
Dryas octopetala	1	2	7	13
Elymus alaskanus	1	1	1	3
Empetrum nigrum	3	7	28	68
Epilobium anagallidifolium	1	1	1	1
Equisetum arvense	1	1	1	2
Equisetum fluviatile	2	4	5	11
Equisetum palustre	2	2	4	5
Equisetum pratense	1	1	2	6

Species/Taxon	Mountains	Transects	Stations	Plots
Eriophorum scheuchzeri	1	1	1	1
Eriphorum angustifolium	1	1	1	1
Eriphorum latifolium	1	1	1	3
Euphrassia frigida	1	1	1	1
Festuca ovina	1	1	2	2
Festuca rubra	1	1	2	5
Festuca vivipara	2	2	4	8
Gnaphalium supinum	1	1	1	1
Hieracium sect *Alpina*	3	5	8	8
Hieracium sect *Oreadea*	1	1	1	2
Hieracium sect *Subalpina*	1	1	1	1
Hieracium sp.	2	4	5	7
Huperzia selago	3	6	13	23
Juncus arcticus	1	1	1	1
Juncus sp.	2	2	2	2
Juncus trifidus	3	8	20	49
Juniperus communis	2	2	2	2
Kobresia myosuroides	1	1	1	1
Linnaea borealis	1	1	2	2
Loiseleuria procumbens	3	8	21	45
Luzula arctica	1	1	1	1
Luzula arcuata	2	2	4	11
Luzula multiflora	3	7	13	19
Luzula sp.	2	2	2	4
Luzula spicata	1	1	1	1
Luzula sudetica	2	2	2	3
Lycopodium annotinum	1	2	5	7
Lycopodium clavatum	1	1	2	2
Lycopodium sp.	2	2	2	2
Lysimachia europaea	1	1	1	2
Matteuccia struthiopteris	1	1	1	1
Minuartia rubella	1	1	1	1
Molinia caerulea	2	4	11	32
Oxyria digyna	3	7	11	14
Parnassia palustris	1	1	2	2
Pedicularis flammea	1	1	1	1
Pedicularis hirsuta	3	4	8	13
Pedicularis lapponica	2	5	8	12
Pedicularis oederi	1	1	1	1
Petasites frigidus	1	1	1	2
Phyllodoce caerulea	3	7	14	29
Pilosella floribunda	1	1	1	1
Pilosella officinarum	1	1	1	1
Pilosella sp.	2	2	2	4
Poa supina	1	1	2	3
Polygonum viviparum	2	2	5	10
Potentilla crantzii	1	1	2	4
Potentilla nivea	1	1	1	1
Pyrola media	1	1	1	1
Pyrola rotundifolia	1	1	1	1
Ranunculus acris	1	2	2	5

Species/Taxon	Mountains	Transects	Stations	Plots
Ranunculus auricomus	1	1	2	2
Ranunculus glacialis	3	5	19	42
Ranunculus nivalis	1	1	1	1
Ranunculus pygmaeus	2	2	3	6
Ranunculus sulphureus	2	2	2	2
Rhodiola rosea	1	1	3	6
Rubus chamaemorus	3	5	7	13
Rumex acetosa	3	5	7	8
Rumex sp.	1	1	1	1
Sagina caespitosa	1	1	1	1
Salix glauca	3	4	5	7
Salix herbacea	3	11	45	144
Salix lapponicum	2	2	2	4
Salix myrsinites	1	1	1	2
Salix polaris	3	9	20	53
Salix reticulata	3	3	8	21
Salix sp.	2	3	3	3
Saxifraga cernua	1	1	1	1
Saxifraga cespitosa	1	1	1	2
Saxifraga foliolosa	1	1	1	1
Saxifraga oppositifolia	2	2	2	2
Saxifraga paniculata	1	1	1	2
Saxifraga rivularis	1	1	2	3
Saxifraga stellaris	1	1	2	2
Selaginella selaginoides	1	1	1	1
Sibbaldia procumbens	3	7	14	25
Silene acaulis	2	2	5	5
Solidago virgaurea	1	1	2	2
Taraxacum sect *Crocea*	2	2	2	4
Taraxacum sp.	2	3	4	5
Thalictrum alpinum	1	1	1	1
Vaccinium myrtillus	3	5	10	22
Vaccinium sp.	1	1	1	1
Vaccinium uliginosum	3	7	11	21
Vaccinium vitis-idaea	3	9	30	82
Vahlodea atropurpurea	1	2	2	3
Veronica alpina	3	4	7	10
Vicia cracca	1	1	1	1
Vicia sepium	1	1	1	1
Viola biflora	3	6	7	11
